# Natural selection on body size is mediated by multiple interacting factors: a comparison of beetle populations varying naturally and experimentally in body size

**DOI:** 10.1002/ece3.1

**Published:** 2011-09

**Authors:** Angela R Amarillo-Suárez, R Craig Stillwell, Charles W Fox

**Affiliations:** 1 Departmento de Ecología y Territorio, Pontificia Universidad JaverianaTransv. 4 No. 42-00, piso 8, Bogotá, Colombia; 2 Department of Biology and Biochemistry, University of HoustonHouston, Texas 77204; 3 Department of Entomology, University of KentuckyLexington, Kentucky 40546-0091

**Keywords:** Artificial selection, body size, fitness, scramble competition, seed beetle, *Stator limbatus*

## Abstract

Body size varies considerably among species and among populations within species, exhibiting many repeatable patterns. However, which sources of selection generate geographic patterns, and which components of fitness mediate evolution of body size, are not well understood. For many animals, resource quality and intraspecific competition may mediate selection on body size producing large-scale geographic patterns. In two sequential experiments, we examine how variation in larval competition and resource quality (seed size) affects the fitness consequences of variation in body size in a scramble-competing seed-feeding beetle, *Stator limbatus*. Specifically, we compared fitness components among three natural populations of *S. limbatus* that vary in body size, and then among three lineages of beetles derived from a single base population artificially selected to vary in size, all reared on three sizes of seeds at variable larval density. The effects of larval competition and seed size on larval survival and development time were similar for larger versus smaller beetles. However, larger-bodied beetles suffered a greater reduction in adult body mass with decreasing seed size and increasing larval density; the relative advantage of being large decreased with decreasing seed size and increasing larval density. There were highly significant interactions between the effects of seed size and larval density on body size, and a significant three-way interaction (population-by-density-by-seed size), indicating that environmental effects on the fitness consequences of being large are nonadditive. Our study demonstrates how multiple ecological variables (resource availability and resource competition) interact to affect organismal fitness components, and that such interactions can mediate natural selection on body size. Studying individual factors influencing selection on body size may lead to misleading results given the potential for nonlinear interactions among selective agents.

## Introduction

Body sizes of organisms vary considerably among species and among populations within species (Peters 1983; Blanckenhorn et al. 2006). For example, body size commonly increases with latitude in animals (Blanckenhorn et al. 2006; Chown and Gaston 2010), and these clines can evolve rapidly following colonization of new geographic regions (e.g., new continents; Huey et al. 2000). Understanding the sources of selection that generate such repeatable and rapidly evolving geographic patterns of body size is important because size has a profound effect on almost all physiological and life-history traits of organisms (Peters 1983; Schmidt-Nielsen 1984; Brown et al. 2004).

If variation in body size among populations, or among species within taxa, is produced by selection then it is necessarily due to variation in the balance between sources of selection favoring large body size and other sources of selection favoring early maturation (and thus indirectly small size) or directly favoring small size (Berger et al. 2006; Kingsolver and Diamond 2011). For example, in insects fecundity selection favors large females (Preziosi et al. 1996) and many sources of sexual selection favor large males, whereas selection favoring early reproduction and reduced larval mortality favor small size (e.g., because taking longer to develop to a larger size increases the risk of mortality due to predation; Fairbairn 1997; Blanckenhorn 2000, 2005; Stillwell et al. 2010). Variation across space in any one of these sources of selection could generate variation in body size (e.g., Fox and Czesak 2006), but which specific sources of selection are geographically variable and responsible for generating geographic variation in body size is poorly understood. Clines in climate, especially temperature and seasonality, are generally argued to mediate selection in such a way as to produce latitudinal clines in size (e.g., Boyce 1978; Lindstedt and Boyce 1985), but it remains unclear if and how climate mediates selection on size (Stillwell et al. 2008; Ho et al. 2010; Stillwell 2010). Alternatively, many ecological variables, such as food availability, population density, and interspecific interactions, vary spatially. However, few studies have examined the extent to which ecological variables mediate selection on body size and thus potentially explain biogeographic patterns in size (Ho et al. 2010).

Geographic variation in resource availability and interspecific or intraspecific competition are likely important sources of variation in selection on body size of many animals, especially those with limited dispersal capacity (e.g., most insect larvae). For example, in *Drosophila*, variation in larval crowding (larval competition) is common among natural populations (Atkinson 1979; Grimaldi and Jaenike 1984; Thomas 1993). Increases in larval density generally increase mortality, reduce growth rate, and reduce body size at maturation and thus have a huge impact on adult fitness (Prout and McChesney 1985; Joshi 1997). These effects of competition on larval development likely vary with larval size, such that larval competition can mediate selection on body size. Indeed, experimental evolution studies with *D. melanogaster*, in which experimental populations are allowed to adapt to different larval competition regimes in the laboratory, show that body size can evolve to be larger at lower larval densities (Roper et al. 1996; but see Santos et al. 1997). Similarly, large evolutionary responses to variation in larval density have been observed in other experimental evolution studies (e.g., Tucic et al. 1997). Intraspecific competition thus appears to be an important mediator of selection generating variation in body size of many insects.

However, despite clear evidence that selection on body size varies with resource availability and resource competition, how competition mediates selection on body size is poorly understood; for example, how do resource availability and competition independently versus interactively affect selection, and which fitness components are most affected? For insects that use discretely packaged resources, such as parasitoids that use a single host larva, or seed-feeding insects that cannot move among seeds, resource size constrains growth (Hardy et al. 1992; Mackauer and Chau 2001; Traynor and Mayhew 2005; Bonal and Muñoz 2009) by either limiting the resources needed for growth or by mediating the intensity of larval competition. These effects on growth can in turn mediate selection on body size. For example, in seed-feeding beetles, species or populations adapted to small seeds are usually genetically smaller-bodied than those adapted to large seeds (Dickason 1960; Center and Johnson 1974; Toquenaga and Fuji 1990; Amarillo-Suárez and Fox 2006). Larger body size may commonly evolve in response to an increase in host size, because most insects are scramble competitors and large hosts provide more resources (i.e., more food) to be spread among competitors. However, in some species (e.g., *Callosobruchus maculatus*) larger individuals are better contest competitors (Messina 1991) and evolve to be large-bodied when in small seeds (Fox et al. 2004; Messina 2004). The evolution of body size in response to variation in host size thus varies among species, in part mediated by the type of competition strategy employed by the insects. However, despite clear documentation of the fitness consequences of variation in host size, and clear evidence that variation in host size influences the evolution of insect body size (Stillwell et al. 2007a), no study has teased apart the relative contribution of resource size versus larval competition to variation in selection on body size.

To evaluate how host size and larval competition affect selection on adult body size, body size must be manipulated and the fitness consequences of variation in body size quantified across a range of host sizes and larval densities. Body size can be altered by manipulating resource availability or by manipulating larval density, but both of these approaches are problematic because numerous growth and life-history traits are affected when larval stress is manipulated (Hardy et al. 1992; Petitt and Wietlisbach 1993; Joshi 1997; Amarillo-Suárez and Fox 2006). Likewise, comparing the fitness consequences of variation in host size and larval density among populations adapted to different sized hosts is problematic because these populations will differ in a number of traits that may be uncorrelated with size but which affect response to these variables. An alternative approach is to compare the fitness consequences of variation in host size and larval density among groups of organisms that vary in body size created by imposing artificial selection directly on body size (Teuschl et al. 2007). Artificially selected lines certainly differ in a suite of traits that evolve through genetic correlations with body size, but these traits are genetically correlated with size, mediating indirect selection on body size, and are thus relevant to studies of the fitness consequences of variation in body size.

Experiments on *D. melanogaster* that have compared flies selected for large versus small body size indicate that the balance between natural selection for large size versus natural selection for early maturation (and thus small size) varies with larval density, with smaller flies having the highest fitness at higher densities (Santos et al. 1992, 1994; Partridge and Fowler 1993; Cortese et al. 2002). This suggests that density-dependent selection will generate considerable geographic variation in body size associated with variation in larval density. However, few studies have used artificial selection to examine the relative fitness of large versus small size across a gradient of larval densities, and all of these studies have been conducted on either *Drosophila* or dung flies (Reim et al. 2006; Teuschl et al. 2007). Furthermore, no study has teased apart the relative effects of resource availability versus larval density (and associated larval interactions). Doing so requires the simultaneously evaluation of relative fitness of large- versus small-sized animals across both a gradient in resource availability (e.g., host size) and larval density.

In this study, we examine how variation in larval competition and host seed size affect the fitness consequences of variation in body size in the seed beetle, *Stator limbatus* (Coleoptera: Chyrsomelidae: Bruchinae). *Stator limbatus* is a generalist seed-feeding beetle that is widely distributed from northern South America to the southwestern United States (Johnson and Kingsolver 1976; Johnson et al. 1989). Larvae develop inside seeds and are restricted to the seed upon which their egg is laid, subjecting them to substantial larval scramble competition (Mitchell 1977). *Stator limbatus* uses a large number of host species that vary considerably in the sizes of seeds they produce. Though *S. limbatus* develop with multiple larvae within a seed, the effect of larval competition is less in larger seeds (Fox et al. 1996). Body size of *S. limbatus* also varies considerably among populations (Stillwell et al. 2007a). This variation is partially genetically based (Amarillo-Suárez and Fox 2006) and is correlated with among-population variation in host plant seed size; beetle size increases with increasing seed size (Stillwell et al. 2007a).

Here we compare the fitness of variation in *S. limbatus* body size across a range of larval densities and a range of seed sizes. We compare two types of variation in body size. First, we compare three field-collected populations that represent the natural range of variation in size, each reared on seeds of three sizes at a range of densities. Second, we use artificial selection to create replicate lines of large-, medium-, and small-bodied populations of females, and compare these at the same range of densities and host sizes. For both experiments, we examine variation among lines in the effects of larval density and seed size on larval survivorship, development time, and adult body mass at maturation to evaluate the relative fitness consequences of being larger versus smaller when reared on different size hosts and at different larval densities. We predict that the relative fitness advantage of being large will decline with decreasing seed size and increasing larval density, matching the evolved (e.g., Stillwell et al. 2007a) and plastic (Fox et al. 1996; Amarillo-Suárez and Fox 2006) effects of these variables on body size of *S. limbatus*.

## Methods

### Natural history of *Stator limbatus*

*Stator limbatus* (Horn) (Coleoptera: Chrysomelidae: Bruchinae) is a seed-feeding beetle with a broad geographic distribution ranging from the northwest of Argentina in South America to the southwest of the United States in North America (Johnson and Kingsolver 1976; Johnson et al. 1989). *Stator limbatus* feeds on seeds of > 70 species of legume trees throughout its wide distribution, though populations at each locality usually have access to just a few species. Most hosts are native (∼ 50 spp.), but many are aliens (> 20 spp.; Morse and Farrell 2005a, Morse 2005b).

The life cycle of *S. limbatus* revolves around seeds. Females oviposit directly onto mature seeds inside of seed pods. First instar larvae hatch from eggs and burrow directly into the seed. Egg-to-adult development takes place entirely inside seeds. Adults emerge 28-30 days later at 28°C. In the laboratory, oviposition in southwestern United States desert populations starts 12-48 h after emergence. Oviposition in Colombian populations starts ∼48 h after emergence.

## Study Populations

The populations used in this study were collected at three different localities from three different host species that vary considerably in the sizes of seeds they produce. We collected beetles from the small-seeded *Pseudosamanea guachapele* (Anapoima, Cundinamarca, Colombia, South America, 4°31'13″N; 74°32'22″W) in December 2002; the medium size seeds of *Acacia greggii* (Oracle, Pinal Co., Arizona, United States, 32°36'39″N; 110°46'13″W) in August 2002; and the large-seeded *Acacia berlandieri* (Del Rio, Texas, United States, 29°28'31″N; 100°59'21″W) in August 2003. Oracle beetles are on average 8% smaller (body mass) than Del Rio beetles, and Anapoima beetles are 52% smaller than Del Rio beetles. *Acacia greggii* seeds are 15-20% smaller than *A. berlandieri* seeds, and *P. guachapele* seeds are 60% smaller than *A. berlandieri* seeds. Field-collected populations (Del Rio, Oracle, and Anapoima) differ in a variety of growth and life-history traits other than mean body mass. Many of these differences are likely a consequence of adaptation to different host species and to seeds of different size (Amarillo-Suarez and Fox 2006).

We collected mature seed pods from at least 20 trees at each locality. Emerging adults (>200 from each population) were used to establish laboratory colonies. Each colony was maintained in the laboratory at >100 families per generation at 28°C, 15:9 light:dark for at least nine generations before initiating experiments. Because survivorship of all populations is very high on *A. greggii* seeds (Amarillo-Suarez and Fox 2006) we maintained all colonies on this host prior to beginning the experiment, to avoid any unintentional selection. The use of a common host eliminated host-associated maternal effects that can confound population differences in growth and body size (Fox et al. 1995). All beetles in the colonies were raised to adult at one larva per seed to eliminate larval competition and thus reduce maternal density effects on offspring traits (inherited environmental effects; Fox 1997; Fox and Savalli 1998).

## Selection Lines

Because variation among populations in their responses to larval density and seed size is confounded by the evolutionary histories of these natural populations (they are adapted to different hosts), we created lines of beetles that differed in mean body size. Using artificial selection, we created two replicates each of large (UP line), small (DOWN line), and medium size (CONTROL line) beetles. All selection lines were created from the Oracle population and thus differences between the lines can only be a consequence of selection for differences in body size and not a consequence of different evolutionary histories with respect to seed size or larval density.

Details of creation of the selection lines are presented in Moya-Laraño et al. (2007). In short, large and small beetles were created by imposing artificial selection on female body size (two replicates each of an UP and a DOWN line). These selection lines were paired with unselected CONTROL lines, which were created by mating randomly chosen offspring (two replicate CONTROL lines). For the selected lines (UP and DOWN) 25 families of beetles were raised per generation, each with 10 offspring (250 total offspring), from which the 25 largest (UP lines) or smallest (DOWN lines) females were selected for the next generation. Emerging females were weighed within 12 h of adult emergence, and then paired with a randomly chosen male from the same line. Females were allowed to lay eggs until they laid one egg on each of >10 *A. greggii* seeds. Ten of these eggs from each female were raised for the next generation. In the CONTROL lines, two random eggs were selected from every female such that no selection was imposed on body size.

Selection was imposed for nine generations, after which beetles were raised for two generations without selection to eliminate any maternal or environmental effects. At the end of selection, UP beetles were 30% larger than CONTROL beetles, and DOWN beetles were 40% smaller than CONTROL beetles.

## Experimental Design

We conducted two independent factorial experiments to examine how host size and larval competition affect selection on adult body size. Both experiments were identical except for the study populations that were used and that they were run sequentially rather than simultaneously (and are thus treated as separate experiments rather than components of a single experiment). Experiment 1 compared the three natural populations of *S. limbatus* that naturally differed in body size (the largest beetles from Del Rio, the medium-size beetles from Oracle, and the smallest beetles from Anapoima). Experiment 2 compared the three artificially selected lines of beetles that differed in body mass (UP, CONTROL, DOWN lines representing the largest, medium, and smallest sized beetles, respectively).

*Experimental overview*: Pairs of beetles from each of the three populations/lines (except Anapoima; see below) were allowed to oviposit on clean seeds (seeds bearing no eggs and that had not been used by any larva) of three different sizes (large, medium, and small) and were reared to adult at four different densities (one, two, three to four, and five to six eggs per seed). Anapoima females do not lay eggs when enclosed with just one male and were thus mated in groups of three females and two males. Large- and medium-sized seeds were *A. greggii* seeds sorted by diameter using a sieve. Average mass for large and medium seeds were 2039 ± (SD) 10 mg and 771 ± 6 mg, respectively. *Acacia greggii* seeds that had not developed normally (e.g., aborted/abnormal) were not used. For the smallest seed class we used seeds of *P. guachapele* that had an average mass of 346 ± 3 mg. Thus, small seeds differed from large and medium seeds in both size and species. This was unavoidable because few *A. greggii* seeds are small enough for this treatment, and those few are typically undeveloped or aborted seeds; we discuss (below) how this affects our results. Only eggs that hatched were considered when establishing larval density treatments.

*Experimental details*: Twelve hours after emergence, each virgin female from each line/population was mated to a single virgin male from the same population/line (these matings were in groups of three females plus two males for the Anapoima population), and randomly assigned to a seed size treatment. Each mated pair of beetles was confined with one, two, four, or eight clean seeds to obtain one, two, three to four, or five to six eggs per seed; pairs of beetles (or groups for the Anapoima population) were provided with eight seeds (to obtain a density of one egg per seed), four seeds (to obtain two eggs per seed), two seeds (to obtain three to four eggs per seed), or two seeds (to obtain five to six eggs per seed). Seeds were examined every 24 h until the predefined number of eggs per seed were laid. Excess eggs laid on the seeds were scraped off with a pair of forceps. This ensured that larval density treatments were largely defined by the experimenters, and not by the beetles themselves. Unhatched eggs were discarded before assigning density treatments. Seeds bearing eggs were placed in a growth chamber at 28°C, L: D 15:9, at one seed per dish.

*Summary of treatments and sample sizes*: A total of four density treatments were established on three sizes of seeds per population per line (one, two, three to four, and five to six larvae/seed). We raised larvae from a total of 3654 hatched eggs (1683 seeds) for Experiment 1 (comparing natural populations) and 7222 hatched eggs (3321 seeds), roughly evenly divided among the two replicate sets of selection lines for Experiment 2 (comparing selected lines).

We recorded larval survivorship (from egg hatch to adult emergence) and development time (from egg hatching to adult emergence) of all surviving beetles. All emerging adult beetles were weighed on electronic balances (Mettler Toledo AT261 Delta range, Columbus, Ohio, USA) to 0.01 mg within 12 h of emergence.

## Statistical Analyses

All statistical analyses were done with SAS version 9.1 (SAS Institute, Cary, North Carolina, USA) using analysis of variance (ANOVA; SAS PROC GLM). To control for nonindependence among siblings, beetle family was treated as the lowest level of independence; beetle family (nested within population ´ treatment interaction) was included in all analyses and used as the denominator mean square for *F*-tests. Hatch-to-adult survivorship was analyzed using a logistic regression.

For our statistical models, population/selection line, replicates (nested within selection lines, as in McCabe and Partridge 1997; Reeve et al. 2000), density, seed size, and sex were treated as fixed main effects. We included the main effects and all possible interactions between population/selection line and treatments in the ANOVAs. Both sex and a sex-by-population interaction were included in our models (for development time and body size) to account for sexual dimorphism and population variation in sexual dimorphism (Stillwell et al. 2007a; Stillwell and Fox 2007).

To test the relative fitness consequences of being larger versus smaller when reared on different size hosts and at different larval densities, we focused on the interactions between (1) population (selection line) and seed size, and between (2) population and rearing density, and (3) the three-way population-by-seed size-by-larval density interaction. However, interactions between factors in an ANOVA are dependent on scale because ANOVA measures changes in the linear distance between treatment means. Interactions can thus be misleading when there is a very large effect of one of the factors (e.g., seed size or larval density) (Stanton and Thiede 2005; Stillwell et al. 2007b). For example, a difference in body mass of 2 mg between selection lines has different meaning when reared on large seeds than when reared on small seeds because the overall average body size changes with seed size. To remove these scaling effects, we log-transformed the data prior to analysis.

## Results

### Main effects: population/line, seed size, and larval density

#### Experiment 1 (natural populations)

**Figure 1:**
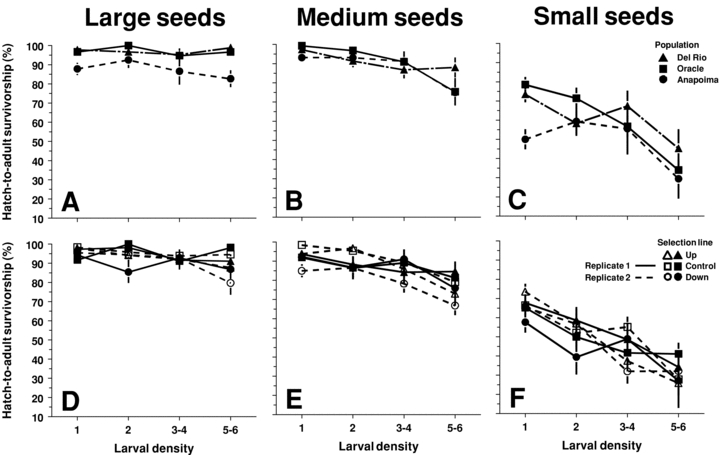
Hatch-to-adult survivorship for Experiment 1 (natural populations; A,B,C) and Experiment 2 (selection lines; D,E,F) of the seed beetle *Stator limbatus* raised on large (A,D), medium (B,E), and small seeds (C,F), at different larval densities (one, two, three to four, and five to six larvae per seed). Contrasts for Del Rio versus Anapoima: 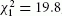, *P* < 0.001; Del Rio versus Oracle: 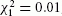, *P* = 0.99; Oracle versus Anapoima: 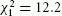, *P* < 0.001. Contrasts for UP versus DOWN: 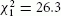, *P* < 0.001; UP versus CONTROL: 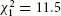, *P* < 0.001; CONTROL versus DOWN 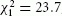, *P* < 0.001. Some error bars are are smaller than the points.

**Figure 3:**
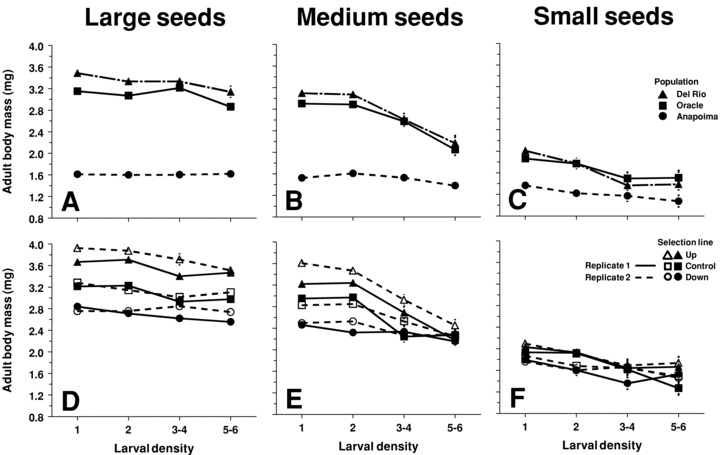
Adult body mass for Experiment 1 (natural populations; A,B,C) and Experiment 2 (selection lines; D,E,F) of the seed beetle *Stator limbatus* raised on large (A,D), medium (B,E), and small seeds (C,F), at different larval densities (one, two, three to four, and five to six larvae per seed). Linear contrasts for Del Rio versus Anapoima: *F*_1,456_ = 750, *P* < 0.001; Del Rio versus Oracle: *F*_1,456_ = 3.87, *P* = 0.049; Oracle versus Anapoima: *F*_1,456_ = 685, *P* < 0.001. Linear contrasts for UP versus DOWN: *F*_1, 979_ = 283, *P* < 0.001; UP versus CONTROL: *F*_1, 979_ = 121, *P* < 0.001; CONTROL versus DOWN: *F*_1, 979_ = 36.9, *P* < 0.001. Some error bars are are smaller than the points.

Hatch-to-adult survivorship, hatch-to-adult development time, and body size at adult emergence varied substantially with seed size, larval density, and among populations (Table 1). Beetles from Anapoima, the smallest-bodied population, had on average (comparing least squares means, removing effects of density and seed sizes) a 10% lower hatch-to-adult survivorship than the Del Rio population (linear contrasts, *P* < 0.001; see figure legends for details of the linear contrasts; Fig. 1A-C). The variance in development time among populations was small and nonsignificant (Table 1). As expected, the large-bodied Del Rio beetles were 75% larger than small-bodied Anapoima beetles and the medium-bodied Oracle beetles were 67% larger than small-bodied Anapoima beetles (*P* < 0.001 for both contrasts; Fig. 3A-C).

**Table 1:** The effects of population, larval density, and seed size (Type III sums of squares) on hatch-to-adult survivorship, hatch-to-adult development time, and body size of the Anapoima (smallest-bodied), Oracle (medium-sized), and Del Rio (largest-bodied) populations of *Stator limbatus*

	Hatch-to-adult survivorship χ*^2^* (df) *P*	Hatch-to-adult development time *F* (df) *P*	Adult body mass *F* (df) *P*
Population	19.8 (2)***	2.32 (2) *ns*	393 (2)***
Density	29.5 (3)***	11.57 (3)***	46.3 (3)***
Seed size	177 (2)***	193 (2)***	384 (2)***
Sex^1^	-	4.84 (1)*	238 (1)***
Sex x population	-	0.04 (2) *ns*	14.3 (2)***
Population x density	14.4 (6)*	0.46 (6) *ns*	2.97 (6)**
Population x seed size	8.20 (4) *ns*	1.41 (4) *ns*	16.9 (4)***
Density x seed size	16.7 (6)*	0.29 (6) *ns*	9.19 (6)***
Population x density x seed size	7.56 (12) *ns*	0.73 (12) *ns*	1.22 (12) *ns*
Family	(517)	(456)	(456)

* *P*≤ 0.05

** *P*≤ 0.01

*** *P*≤ 0.001; *ns*, *P* > 0.05.

1 Larvae cannot be sexed until the adult stage.

**Figure 2:**
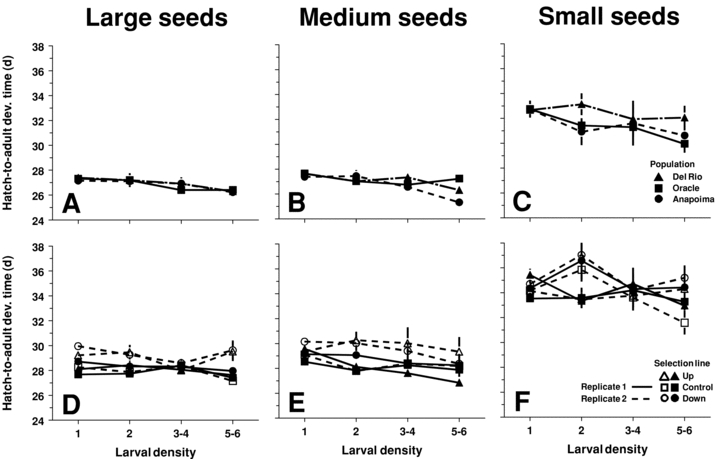
Hatch-to-adult development time for Experiment 1 (natural populations; A,B,C) and Experiment 2 (selection lines; D,E,F) of the seed beetle *Stator limbatus* raised on large (A,D), medium (B,E), and small seeds (C,F) at different larval densities (one, two, three to four, and five to six larvae per seed). The variance in development time among natural populations was small and nonsignificant (Table 1) so no contrasts are presented. Linear contrasts for UP versus DOWN: *F*_1, 979_ = 6.05, *P* = 0.014; UP versus CONTROL: *F*_1,979_ = 7.40, *P* = 0.006; CONTROL versus DOWN: *F*_1, 979_ = 24.1, *P* < 0.001. Some error bars are are smaller than the points.

Survivorship increased substantially with increasing seed size and decreasing larval density (Fig. 1A-C). The seed size effect was probably due to the difference in host species used for the small (*P. guachepele*) versus large and medium (*A. greggii*) seeds; the seed size effect became nonsignificant after eliminating the small seeds from the analysis (*P* = 0.97). Development time increased significantly with decreasing seed size, and decreased (slightly) with increasing density (Figure 2A-C). Like the seed size effect on larval survival, the seed size effect on development time was probably due primarily to differences between the seed species rather than seed size *per se* as the seed size effect became nonsignificant after eliminating small seeds from the analysis (*P* = 0.54). As expected, body size at emergence decreased with both decreasing seed size and with increasing larval density (Fig. 3A-C). In contrast to larval survival and development time, the seed size effect was not due to just the small seeds; the effect remained highly significant even after eliminating small seeds from the analysis (*F*_1,333_ = 122, *P* < 0.001).

#### Experiment 2 (selection lines)

Survivorship, development time, and body size varied considerably among the selection lines, with seed size, and with larval density (Table 2). The DOWN lines (the smallest beetles) had a 6% and 5% lower hatch-to-adult survivorship compared to the CONTROL and UP lines, respectively (*P* < 0.001 for the two contrasts; Fig. 1D-F). Survivorship increased with increasing seed size, an effect that persisted after eliminating small seeds from the analysis (Fig. 1D-F; 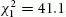, *P* < 0.001). Survivorship also decreased with increasing larval density (Fig. 1D-F), similar to the effect observed in Experiment 1.

**Table 2:** The effects of selection line, larval density, and seed size (Type III sums of squares) on hatch-to-adult survivorship, hatch-to-adult development time, and body size of the Up, Down, and Control lines of *Stator limbatus*

	Hatch-to-adult survivorship χ*^2^* (df) *P*	Hatch-to-adult development time *F* (df) *P*	Adult body mass *F* (df) *P*
Line	27.2 (2)***	11.3 (2)***	125 (2)***
Density	119 (3)***	4.43 (3)**	67.9 (3)***
Seed size	804 (2)***	388 (2)***	966 (2)***
Replicate (Line)	0.03 (3) *ns*	8.33 (3)***	4.80 (3)**
Sex^1^	-	5.18 (1)*	25.2 (1)***
Line x density	11.5 (6) *ns*	1.45 (6) *ns*	4.73 (6)***
Line x seed size	5.72 (9) *ns*	0.98 (9) *ns*	3.58 (9)**
Density x seed size	6.73 (6) *ns*	1.02 (6) *ns*	9.22 (6) ***
Line x density x seed size	13.9 (12) *ns*	1.97 (12)*	2.03 (12)*
Family	(1066)	(979)	(979)

* *P*≤ 0.05

** *P*≤ 0.01

*** *P*≤ 0.001; *nsP* > 0.05.

1 Larvae cannot be sexed until the adult stage.

Hatch-to-adult development time varied significantly among the selection lines, but this variation was quite small (1-3%; Fig. 2D-F; Table 2). Development time increased with decreasing seed size and, in contrast to experiment 1, this effect remained highly significant after eliminating the smallest seeds from the analysis (*F*_1,660_ = 700, *P* < 0.001). Although variation in development time among densities was significant (Table 2), the decline in development time with larval density was less consistent among lines and seed size treatments than observed in Experiment 1 (Fig. 2D-F).

The selection lines differed considerably in body size: UP beetles were 12% larger than CONTROL beetles and the CONTROL beetles were 9% larger than the DOWN beetles (*P* < 0.001 for all contrasts; Fig. 3D-F). Body size increased with increasing seed size (Table 2), an effect that remained highly significant after eliminating small seeds from the analysis (*F*_1,660_ = 1913, *P* < 0.001). Body size also increased with decreasing larval density (Table 2; Fig. 3D-F).

### Population/line-by-density interactions

We expected that the relative advantage of being larger would be greatest at the lowest larval densities, where beetles have adequate resources to reach large body size. This should be detected in our analyses as significant population-by-density interactions for survivorship, development time, and/or body size at emergence.

#### Experiment 1 (natural populations)

Our data indicate that density affects the relative variation among populations in larval survivorship and body size at emergence (there were significant population-by-density interactions for larval survivorship and adult body mass, but not for development time; Table 1). However, support for our prediction is mixed. For example, the relative difference in hatch-to-adult survivorship between the largest-bodied population (Del Rio) and the smallest-bodied population (Anapoima) was greatest at the lowest and the highest larval densities (14% and 19%, respectively) and smallest at the intermediate densities (two larvae = 0.5%; three to four larvae = 6%), inconsistent with the prediction that the relative fitness of large beetles declines with density (*P* = 0.05; Fig. 1A-C). In contrast, the body size of larger beetles declined more rapidly with density than did the body size of smaller beetles (Fig. 3A-C), consistent with our prediction.

#### Experiment 2 (selection lines)

Consistent with the results for the natural populations in Experiment 1, there was no evidence that the relative advantage of being larger was greater at lower larval densities for either hatch-to-adult survivorship or development time (Table 2; Figs. 1D-F and 2D-F). However, as in the natural populations, larger beetles suffered a greater proportional decline in body size with increasing larval density. For example, the relative difference between the UP and DOWN lines declined with increasing larval density, from 24% at low density to 14% at high density (Table 2; Fig. 3D-F; percentage differences based on least-square means controlling for seed size effects).

### Population/line-by-seed size interactions

In nature, populations of *S. limbatus* collected from smaller seeds are smaller bodied, even after many generations of laboratory rearing. We thus predicted that the relative advantage of being large would decline with decreasing seed size.

#### Experiment 1 (natural populations)

We found no evidence that the relative difference in larval survival between larger- and smaller-bodied populations varied with seed size (nonsignificant population-by-seed size interactions for survivorship; Table 1). The population-by-seed size interaction was also nonsignificant for development time, both before and after deleting the smallest seeds (*P. guachepele*) from the analysis. In contrast, there was a highly significant population-by-seed size interaction for body size (*P* < 0.001; Fig. 3A-C); the relative difference in body size between large and small-bodied populations was greatest on the largest seeds and smallest on the smallest seeds (compare among panels A, B, and C of Fig. 3), consistent with the prediction that larger beetles suffer a greater fitness cost to being on small seeds. This population-by-seed size interaction remained highly significant after removing *P. guachapele* seeds from the analysis (*F*_1,333_ = 7.41, *P* < 0.001).

#### Experiment 2 (selection lines)

As with the natural populations, we found no evidence that larger-bodied beetles experienced a greater reduction in survival, or increase in development time, with decreasing seed size; the line-by-seed size interaction was not significant for either hatch-to-adult survivorship or development time (Table 2). However, as in the natural populations, larger beetles suffered a much greater reduction in body size on the largest seeds (Table 2; Fig. 3D-F). For example, body size of DOWN line beetles varied only a little among the three seed sizes (compare open circles in Fig. 3 panels D, E, and F; average difference between large and small seeds is 41%) whereas UP line beetles experienced on average a 50% reduction in body size between the largest and smallest seeds. This population-by-seed size interaction remained highly significant after removing *P. guachapele* seeds from the analysis (*F*_1,660_ = 6.56, *P* < 0.002).

### Seed size-by-larval density interactions and three-way interactions

#### Experiment 1 (natural populations)

The effects of larval density on larval survival and adult body mass, but not development time, changed with seed size (seed size-by-larval density interactions in Table 1; both effects remained highly significant after deleting *P*. *guachapele* seeds, *P* < 0.005). For example, the decline in larval survivorship with increasing larval density was greater when beetles were raised on smaller seeds (Table 1; Fig. 1A-C). Likewise, the decline in body mass with increasing larval density was substantially greater when raised on the smaller seeds (Fig. 3A-C). Thus, the effects of density and seed size on larval survival and adult body size were not strictly additive.

#### Experiment 2 (selection lines)

The effects of larval density on adult body mass varied with seed size (Table 2). The decrease in body mass with increasing larval density was considerably larger on the smaller seeds (Fig. 3D-F). This seed size-by-larval density interaction varied among the selection lines (significant three-way interaction; Table 2). This three-way interaction for body size is easily visualized in the figures (Fig. 3D-F) and mirrors the (non-significant) pattern seen for the natural populations (Fig. 3A-C); the variation among selection lines in the effects of larval density is small when beetles are reared on large seeds, but is more substantial at intermediate and small seeds.

## Discussion

Natural populations of *S. limbatus* vary substantially in body size over their geographic range, and much of this variation is genetically based (Amarillo-Suárez and Fox 2006). As in many parasitic insects (e.g., parasitoids and seed beetles), much of this variation in body size is associated with variation among populations in host size (Amarillo-Suárez and Fox 2006; Stillwell et al 2007a) and is influenced, at least phenotypically, by larval competition (Fox et al. 1996). Here, we examined whether host size and larval competition differentially affect survival and growth of large versus small beetles, and thus affect the relative fitness consequences of being large versus small. Specifically, we predicted that larger beetles would suffer a greater fitness cost, relative to smaller beetles, with both decreasing seed size and increasing larval density. Our results are mixed regarding this prediction. We found that larger beetles experience similar effects of larval competition and seed size on their larval survival and larval development time, compared to smaller beetles (there were only nonsignificant or inconsistent [between experiments] population/line-by-seed size and population/line-by-density interactions). However, we found that larger beetles suffer a much greater reduction in body size (and thus, presumably, all fitness traits, such as fecundity [Honek 1993; Davidowitz 2008], that are influenced by adult size), relative to smaller beetles, in response to increasing larval density and decreasing seed size. We conclude that variation in both larval density and seed size influences the relative fitness advantage of being large-the advantage is reduced at higher density and on smaller seeds-and thus mediates selection on body size by influencing resource acquisition and adult size. However, the effects of density and seed size on selection were not strictly additive; there was a significant three-way interaction (line-by-density-by-seed size in Experiment 2) indicating that the effects of density on the relative fitness consequences of being large depend on seed size, and vice versa.

Our laboratory study could examine only a small number of the wide diversity of variables that likely affect selection on body size, and examined only a subset of the possible components of *S. limbatus* fitness. In this simplified laboratory context, our data indicate that larger beetles are better overall-they typically had higher survivorship, and had very similar development times despite being larger (indicating they have higher growth rates), relative to smaller beetles. However, this will clearly not be so simple in nature; other components of selection, during development or on adults (e.g., selection favors small males during mate finding; Moya-Laraño et al. 2007), or in response to other environmental conditions (such as heat stress [Levins 1969], water availability [Stillwell et al. 2007a], and predation risk [Remmel and Tammaru 2009]), must necessarily balance this selection for large size to produce the distribution of sizes observed in nature. Our current study tells us nothing about these other sources of selection, or other components of fitness. Instead, the key conclusion of our study is just that the balance between all of these various sources of selection on adult body size will shift toward favoring smaller size (all else being equal) at higher larval density and on smaller seeds.

A few studies have examined the consequences of variation in either larval density (Roper et al. 1996; Santos et al. 1997; Tucic et al. 1997; Pérez and García 2002) or resource availability such as host size (Messina 2004) for selection on and/or the evolution of body size. For example, small body size evolves in response to increasing larval competition in *D. melanogaster* (Roper et al. 1996; but see Santos et al. 1997) and in another seed-feeding beetle, *Acanthoscelides obtectus* (Tucic et al. 1997). We found that the relative magnitude of selection favoring large body size of *S. limbatus* declines with increasing density, consistent with these experimental evolution results for *Drosophila* and *A. obtectus*, but not consistent with an experimental evolution study of a different seed-feeding beetle, *C. maculatus* (Messina 2004). The difference is likely in part due to the types of competition (scramble vs. contest) experienced within seeds, and associated differences in the type of selection (density- vs. frequency-dependent). Like *S. limbatus*, *A. obtectus* appears to exhibit primarily scramble competition within seeds, and thus larvae experience density-dependent selection mediated by volume of resources (Szentesi 2003). Consequently, small body size evolves when larval competition is increased (e.g., because smaller seeds have fewer resources; Tucic et al. 1997). In contrast, for *C. maculatus*, increased aggressiveness evolves in response to increased density or small seed size (due to increased frequency of interactions; Smith and Lessells 1985) despite the lower efficiency of exploitation of seeds (Messina 1991). This increase in aggressiveness increases the frequency of contest interactions within seeds, and favors the evolution of large body size due to the competitive advantage in an aggressive contest of being larger (Messina 2004). This effect of competition type on how density mediates selection on body size may be a general rule; in scramble-competing species, increasing larval density leads to either the evolution of small body size, or no evolution in size (Borash and Ho 2001; Sanders et al. 2005; but see Mueller 1997), whereas it is a fairly general observation that increasing contest interactions favors larger body size (Simmons 1987; Briffa and Sneddon 2010).

Many previous studies have shown that host size (seed or host insect size; Dickason 1960; Center and Johnson 1974; Toquenaga and Fuji 1990; Hardy et al. 1992; Petitt and Wietlisbach 1993; Amarillo-Suárez and Fox 2006), and larval density (Colegrave 1995; Cornelissena and Stiling 2008; Yanagi and Tuda 2010) affect both growth and survival of seed beetles. For example, numerous studies of *C. maculatus* have examined larval density or seed effects on development (Guedes et al. 2007; review in Smallegange and Tregenza 2008), and a previous study of *S. limbatus* found very similar effects of larval density to those reported here (Fox et al. 1996). However, in nature both resource patch size (e.g., seed or fruit size) and insect larval density vary simultaneously (Hardy et al. 1992; Mackauer and Chau 2001; Bonal and Muñoz 2009) and may have independent or interactive effects on selection on body size (Vamosi 2005; Guedes et al. 2007). At least one previous study has shown that these two variables have interactive (nonadditive) effects on larval survival (e.g., Toquenaga and Fuji 1990), consistent with our results (we detected a significant density-by-seed size interaction for almost all fitness traits measured). This interaction is not surprising given that both increasing larval density and decreasing host size are basically severely restricting the amount of food larvae have available. However, our study goes one step further and is novel in examining how simultaneous variation in resource patch size (seed size) and larval density affect the fitness consequences of being larger versus smaller (testing for independent vs. interactive effects on fitness consequences of variation in body size). We found that both variables affect selection on body size, and that these effects are interactive and not additive. Our results suggest that examining the fitness consequences of body size along multiple environmental gradients will more accurately reflect how selection operates in nature, where organisms are typically exposed to multiple environmental variables simultaneously.

This study is also novel in comparing fitness consequences of density and host size manipulations simultaneously among populations artificially selected to vary in size and populations that naturally differ in body size. This comparison is important because it informs us whether the natural variation in size has similar fitness consequences to variation in size created by direct selection on body size. Comparing the fitness consequences of variation in host size and larval density among populations naturally adapted to different sized hosts, or different larval densities, is problematic because these populations will differ in a number of traits that may be uncorrelated with size but which affect response to these variables. For example, larval density and/or seed size may influence the evolution of larval mobility and aggressiveness within seeds (Brodeur and Boivin 2004; Messina 2004), which could then covary with body size among populations (as in Messina 2004) and confound our ability to tease apart the fitness consequences of variation in body size from variation in other traits. Traits such as mobility and aggressiveness should not vary among our artificial selection lines, which were created by selecting only on body size, unless these traits are genetically correlated to body size and hence directly relevant to any discussion on the evolution of body size. That our results agree with regard to all of our major conclusions for both the populations naturally varying in size and the populations artificially selected to vary in size gives us confidence that body size per se, or a variable genetically correlated with size, directly affects the fitness consequences of variation in larval density and seed size.

In our experiment, many (Colombian populations) or all (all other populations) of the offspring within a seed were kin, rather than unrelated individuals. This is likely a normal or at least very common situation for most bruchine seed beetles because females must disperse between patches of seeds (fruits) and they commonly lay multiple eggs per seed once finding a fruit (Mitchell 1977). However, competition among nonsibs within seeds must also be common in nature; in the laboratory, females will readily lay eggs on seeds bearing unrelated conspecific eggs (they prefer seeds with low egg loads but will accept seeds with high egg loads when no alternatives are available), and in the field multiple adult females (that necessarily arrived after dispersal from other fruits) are commonly observed inside individual fruits. It is possible that *S. limbatus* larvae respond to related individuals within seeds differently than they would to unrelated individuals-for example, they may become less physically aggressive and more scramble competing-limiting the generality of our results. However, the evidence for kin recognition by adult seed beetles is mixed (Ofuya and Agele 1989; Messina and Tinney 1991), and there is no evidence that the consequences of larval competition within seeds vary in response to relatedness (Smallegange and Tregenza 2008).

One problem with our experimental design is that our manipulation of seed size is confounded with other variables that differ between seed species; specifically, although we used two sizes of seeds within one species (our large and medium seed size treatments are large versus small *A. greggii* seeds), our small seed size treatment consists of seeds of a different species (*P. guachapele*). This was necessary because *A. greggii* seeds are not small enough to encompass the range of seed sizes that occur among *S. limbatus* hosts in nature (the smallest *A. greggii* seeds are typically aborted and unviable seeds). Thus, any differences between small and medium or large seeds could be (indeed, are likely to be) due to species differences rather than, or in addition to, seed size (Amarillo-Suárez and Fox 2006). However, our results regarding the large population/line-by-treatment effects on beetle body size, and the general lack of such interactions on larval survival and larval development time, all remain robust to deletion of the small seed treatment. Consequently, our main conclusion that the relative advantage of large-bodied beetles is greatest when raised on large seeds and at low levels of larval competition is supported regardless of whether or not we include the small seed class.

## Conclusion

Geographic variation in body size of animals is common in nature. This variation in size often exhibits repeatable patterns; for example, body size commonly varies with latitude. Yet, we have a poor understanding of the ecological variables that mediate selection and generate these patterns. In this study, we manipulated one variable (host seed size) known to covary with body size among populations, and another variable (larval density) known to have large effects on development and survival, and examined the consequences of these manipulations for components of fitness in large- and small-bodied seed beetles. Our results demonstrate that seed size mediates selection on *S. limbatus* body size; the relative advantage of being large-bodied increases with increasing seed size, concordant with the geographic pattern observed in this beetle. We also find that the relative advantage of being large-bodied declines with increasing larval competition, especially on small seeds. Our results thus generally indicate that the advantage of larger-bodied organisms versus small-bodied organisms should generally increase when the amount of resources increase (whether due to host size or larval competition).
